# A case report of anaplastic carcinoma with osteoclast-like giant cells arising in the pancreatic body

**DOI:** 10.1093/jscr/rjac288

**Published:** 2022-06-25

**Authors:** Masaki Kitazono, Makoto Fujita, Ayaka Ito, Tomohiro Oyama, Naotaka Ikeda, Mayumi Eguchi, Rikiya Sato, Shuichiro Uchiyama, Ryoichi Toyosaki, Naoya Yokomakura, Toyokuni Suenaga, Hirotake Kusumoto, Koichiro Tsukasa

**Affiliations:** Department of Surgery, Nanpuh Hospital, Kagoshima-city 892-8512, Japan; Division of Medical Support, Nanpuh Hospital, Kagoshima-city 892-8512, Japan; Department of Surgery, Fujita Health University Hospital, Toyoake-city 470-1192, Japan; Department of Surgery, Nanpuh Hospital, Kagoshima-city 892-8512, Japan; Department of Surgery, Nanpuh Hospital, Kagoshima-city 892-8512, Japan; Department of Surgery, Nanpuh Hospital, Kagoshima-city 892-8512, Japan; Department of Surgery, Nanpuh Hospital, Kagoshima-city 892-8512, Japan; Department of Surgery, Nanpuh Hospital, Kagoshima-city 892-8512, Japan; Department of Surgery, Nanpuh Hospital, Kagoshima-city 892-8512, Japan; Department of Surgery, Nanpuh Hospital, Kagoshima-city 892-8512, Japan; Department of Surgery, Nanpuh Hospital, Kagoshima-city 892-8512, Japan; Department of Gastroenterology, Nanpuh Hospital, Kagoshima-city 892-8512, Japan; Department of Gastroenterology, Nanpuh Hospital, Kagoshima-city 892-8512, Japan

## Abstract

The patient is a 58-year-old woman. She was referred to our hospital following a computed tomography scan that revealed a 2-cm tumor-like lesion in the pancreatic body. Endoscopic ultrasound fine-needle aspiration examination revealed a suspected undifferentiated carcinoma with pleomorphic type. The patient was diagnosed with anaplastic carcinoma of the pancreas (ACP) and underwent distal pancreatectomy with lymph nodes dissection. The resected body and tail of the pancreas had a nodular tumor measuring 30 mm in diameter. Histologically, the main lesion of the tumor showed well-differentiated adenocarcinoma, and diffuse proliferation of atypical short spindle cells and round cells accompanied by multinucleated giant cells aggregation was observed around the tubular structure; hence, it was diagnosed with ACP. The postoperative course was uneventful, and the patient was discharged 14 days after the operation. It has already been about 5 years since the surgery, and although the tumor has recurred, the patient is still alive and undergoing chemotherapy.

## INTRODUCTION

Anaplastic carcinoma of the pancreas (ACP) is a rare tumor occurring at a frequency of 2–7% among invasive pancreatic carcinomas [1, 2] and is considered to have a poor prognosis. According to the Japan Pancreas Society, ACP has been classified into four types (spindle cell type, pleomorphic type, giant cell type and osteoclast-like giant cells [OCGC] type) based on its cell morphology. In this report, we describe a case of ACP with OCGCs in a patient who survived for 5 years after the surgery.

## CASE REPORT

**Figure 1:**
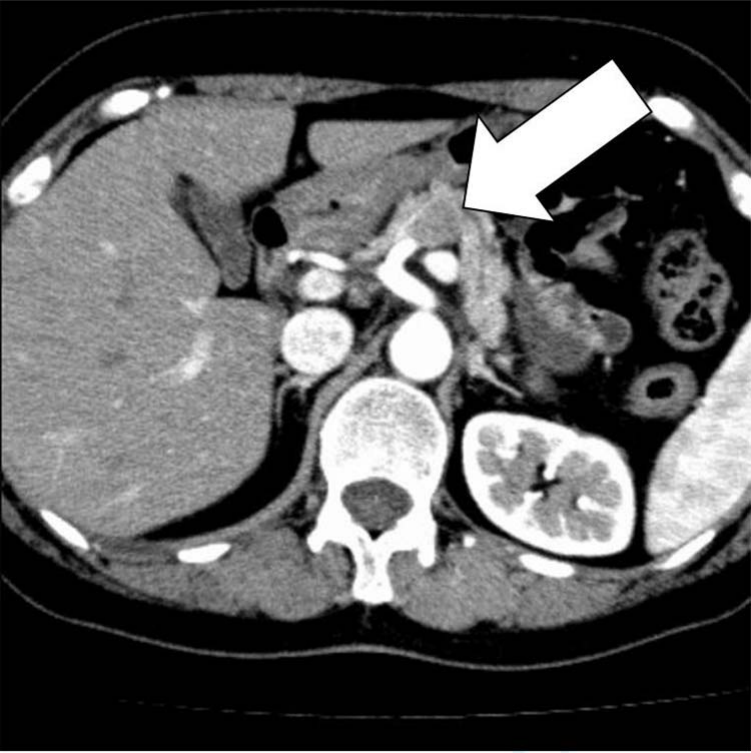
An abdominal CT scan revealed a 2 cm mass with mild contrast effect in the pancreatic body. There was a little tendency to invade the surrounding area, which was different from typical pancreatic ductal carcinoma.

**Figure 2:**
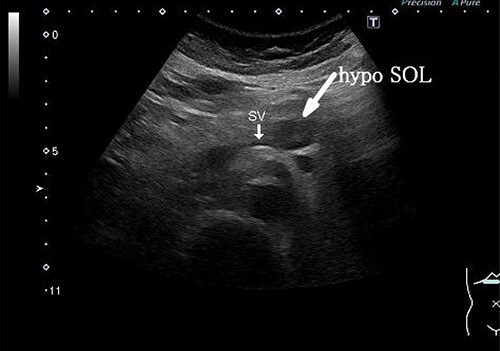
Abdominal US showed a 19.4 x 12.6 mm mass in the pancreatic body. It was a hypoechoic mass with a clear boundary, smooth and homogeneous interior. Pancreatic duct dilation was observed more clearly than around the mass.

A 58-year-old woman began to have back pain and upper abdominal pain and then visited a hospital. Laboratory findings showed mildly elevated total bilirubin 1.3 mg/dl and high amylase 466 IU/l; however, peripheral blood, liver and kidney functions were within normal limits. Tumor markers were within normal limits: CEA 1.1 ng/ml, CA19–9 24.3 U/ml. An abdominal computed tomography (CT) scan revealed a 2-cm mass with mild contrast effect in the pancreatic body. There was a little tendency to invade the surrounding area, which was different from typical pancreatic ductal carcinoma (Fig. 1). Abdominal ultrasonography (US) showed a 19.4 × 12.6 mm mass in the pancreatic body. It was a hypoechoic mass with a clear boundary, smooth and homogeneous interior. Pancreatic duct dilation was observed more clearly than around the mass (Fig. 2).

**Figure 3:**
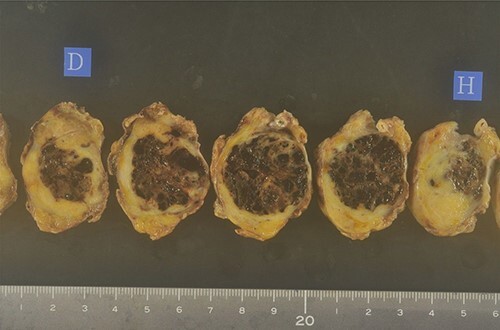
The resected body and tail of the pancreas had a nodular tumor measuring 30 mm in diameter, and cut surface revealed the tumor was surrounded by fibro adipose capsule-like tissue, and the central area was composed of follicular structures that contain brown red-colored fluid.

A biopsy of the pancreatic mass under endoscopic ultrasound fine-needle aspiration revealed atypical foci of cells with multinucleated giant cells suggesting ACP. Distal pancreatectomy with lymph nodes dissection was carried out based on the diagnosis of anaplastic carcinoma of the pancreatic body. The resected body and tail of the pancreas had a nodular tumor measuring 30 mm in diameter, and cut surface revealed the tumor was surrounded by fibro adipose capsule-like tissue, and the central area was composed of follicular structures that contain brown red-colored fluid (Fig. 3).

**Figure 4:**
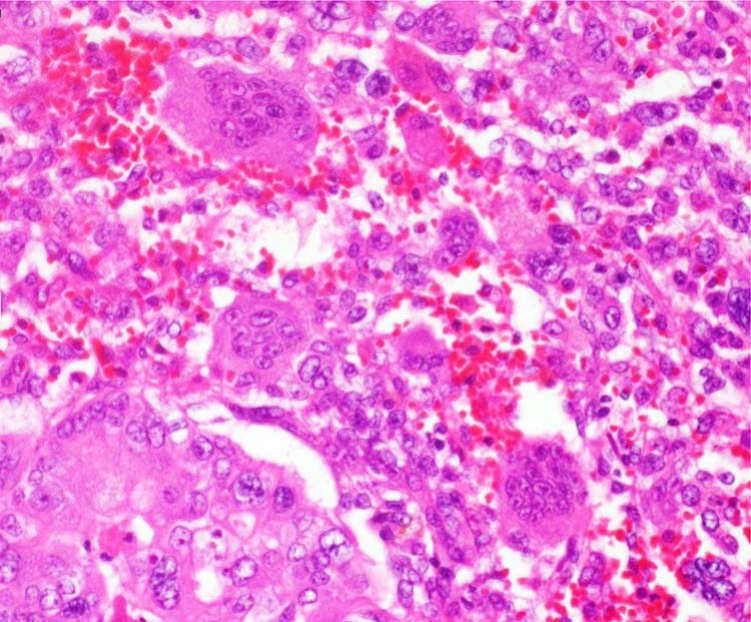
The diffuse proliferation of atypical short spindle cells and round cells accompanied by multinucleated giant cells aggregation was observed around the tubular structure. The giant cells had multiple small round nuclei which gather in the centrocytic portion, resembling OCGCs.

Histologically, the main lesion of the tumor showed proliferation of atypical mucin-producing columnar epithelial cells lining follicle-like or cystically dilated tubular structures representing well-differentiated adenocarcinoma. The diffuse proliferation of atypical short spindle cells and round cells accompanied by multinucleated giant cells aggregation was observed around the tubular structure. The giant cells had multiple small round nuclei, which gather in the centrocytic portion, resembling OCGCs (Fig. 4).

Because of those histopathological findings, the definitive diagnosis of anaplastic carcinoma with OCGCs was made. The patient was discharged on postoperative Day 14 with good progress. Although the patient was followed up periodically in the outpatient clinic, local recurrence at the edge of the remaining pancreas and invasion of the celiac artery were pointed out in the CT scan 4 years and 5 months after the operation. Proton therapy (67.5 GyE) and S-1 chemotherapy were administered, but the tumor spread and was judged to be progressive of disease; thus, the patient is currently undergoing gemcitabine monotherapy.

## DISCUSSION

ACP was first described as pleomorphic carcinoma by Sommers and Meissner in 1954 [3] and is known to be rare with a poor prognosis. According to histology, ACP is mainly classified into three types: spindle cell type, pleomorphic type and giant cell type [4]. The undifferentiated carcinoma with OCGC was reported by Rosai [5] as one of the giant cell types resembling osteoclasts that are prominent. ACP has been reported as aggressive, with a median survival of only 2 months [6–8]. On the other hand, the survival of OCGC type has been reported to range from 4 months to 15 years [9–12]. The symptoms of ACP are often non-specific, such as abdominal pain, malaise, jaundice, weight loss, anorexia and back pain [13]. In total, 60 ACP cases were reported by Hoshimoto *et al*. [14] in Japan with a slight male predominance with a mean age of 61.5 years and 38 cases being males. The main symptoms were abdominal pain (48%), back pain (17%), malaise (13%), fever (10%), jaundice (10%), weight loss (10%) and abdominal discomfort (10%). In our case, the patient had back pain and upper abdominal pain and had been left untreated for some time.

The most frequent site of ACP is head (53%), followed by body–tail (42%) and head–body–tail (5%). The tumors ranged from 1.5 to 24.0 cm with a median of 6.0 cm. Severe anemia (hemoglobin level of <10.0 g/dl), and markedly elevated leukocyte counts (>12 000/mm^3^) were found in 20% of cases, respectively. Elevation of serum CA19-9 levels (>37 U/ml) was observed in 55% of cases. In our case, neither anemia nor high CA19-9 levels were found. In the imaging findings, ACP is usually detected as large, hypervascular and exophytic tumors with widespread necrotic tissue [15]. Tumor or rim enhancement on abdominal CT was observed in 82% of cases. Pancreaticoduodenectomy was performed in 53% of cases, distal pancreatectomy in 40% and total pancreatectomy in 3%, respectively.

The histological subtypes were giant cell type (18%), pleomorphic type (30%), spindle cell type (17%) and OCGC type (35%). In terms of survival, the death rate within 1 year was 51% in all histological types, and the 5-year survival rate was 12%. The percentage of patients who survived for <1 year by histological type consisted of giant cell type (64%), polymorphic cell type (72%), spindle cell type (70%) and OCGC type (11%). OCGC type had a clearly better prognosis than the other types. Hoshimoto *et al*. argue that it is more likely to be resected because the tumor does not grow as fast as other histological types.

## CONCLUSION

Our patient also had a long-term survival, whereas the main reason for this cannot be determined. However, this is a suggestive case in that even ACP can lead to more prolonged survival than expected. The sample size of ACP scarce and effective treatment is not yet known. Therefore, it is necessary to conduct a survey in collaboration with other institutions and accumulate nationwide data to analyze this disease.
